# *Navigate*: a study protocol for a randomised controlled trial of an online treatment decision aid for men with low-risk prostate cancer and their partners

**DOI:** 10.1186/s13063-020-04986-9

**Published:** 2021-01-11

**Authors:** Penelope Schofield, Karla Gough, Amelia Hyatt, Alan White, Mark Frydenberg, Suzanne Chambers, Louisa G. Gordon, Robert Gardiner, Declan G. Murphy, Lawrence Cavdon, Natalie Richards, Barbara Murphy, Stephen Quinn, Ilona Juraskova

**Affiliations:** 1 Department of Psychology, Swinburne University of Technology, Melbourne, Victoria Australia; 2 Behavioural Science Unit, Peter MacCallum Cancer Centre, Melbourne, Victoria Australia; 3 Sir Peter MacCallum Department of Oncology, The University of Melbourne, Parkville, Victoria Australia; 4 Swinburne University of Technology, John Street, Hawthorn, Australia; 5 Department of Nursing, The University of Melbourne, Parkville, Victoria Australia; 6 Department of Urology, Cabrini Institute, Cabrini Health, Malvern, Australia; 7 Department of Surgery, Monash University, Melbourne, Victoria Australia; 8 Faculty of Health, University of Technology Sydney, Sydney, Australia; 9 Health and Wellness Institute, Edith Cowan University, Perth, Australia; 10 Institute for Resilient Regions, University of Southern Queensland, Springfield, Australia; 11 Menzies Health Institute Queensland, Griffith University, Southport, Australia; 12 Population Health Department, Health Economics, QIMR Berghofer Medical Research Institute, Brisbane, Queensland Australia; 13 School of Nursing, Queensland University of Technology (QUT), Brisbane, Queensland Australia; 14 School of Public Health, University of Queensland, Brisbane, Queensland Australia; 15 School of Medicine, University of Queensland, Brisbane, Queensland Australia; 16 Department of Urology, Royal Brisbane & Women’s Hospital, Herston, Queensland Australia; 17 Division of Cancer Surgery, Peter MacCallum Cancer Centre, Melbourne, Victoria Australia; 18 School of Science, RMIT University, Melbourne, Victoria Australia; 19 Department of Psychology, The University of Melbourne, Parkville, Victoria Australia; 20 Faculty of Health, Deakin University, Bundoora, Victoria Australia; 21 Department of Health Science and Biostatistics, Swinburne University of Technology, Melbourne, Victoria Australia; 22 School of Psychology, Faculty of Science, Centre for Medical Psychology and Evidence-based Decision-making (CeMPED), University of Sydney, Sydney, New South Wales Australia

**Keywords:** Low-risk prostate cancer, Decision aid, Management decision, Treatment decision, Active surveillance, RCT, Distress, Quality of life

## Abstract

**Background:**

Active surveillance (AS) is the disease management option of choice for low-risk prostate cancer. Despite this, men with low-risk prostate cancer (LRPC) find management decisions distressing and confusing. We developed *Navigate*, an online decision aid to help men and their partners make management decisions consistent with their values. The aims are to evaluate the impact of *Navigate* on uptake of AS; decision-making preparedness; decisional conflict, regret and satisfaction; quality of illness communication; and prostate cancer-specific quality of life and anxiety. In addition, the healthcare cost impact, cost-effectiveness and patterns of use of Navigate will be assessed. This paper describes the study protocol.

**Methods:**

Three hundred four men and their partners are randomly assigned one-to-one to *Navigate* or to the control arm. Randomisation is electronically generated and stratified by site. *Navigate* is an online decision aid that presents up-to-date, unbiased information on LRPC tailored to Australian men and their partners including each management option and potential side-effects, and an interactive values clarification exercise. Participants in the control arm will be directed to the website of Australia’s peak national body for prostate cancer. Eligible patients will be men within 3 months of being diagnosed with LRPC, aged 18 years or older, and who are yet to make a treatment decision, who are deemed eligible for AS by their treating clinician and who have Internet access and sufficient English to participate. The primary outcome is self-reported uptake of AS as the first-line management option. Secondary outcomes include self-reported preparedness for decision-making; decisional conflict, regret and satisfaction; quality of illness communication; and prostate cancer-specific quality of life. Uptake of AS 1 month after consent will be determined through patient self-report. Men and their partners will complete study outcome measures before randomisation and 1, 3 and 6 months after study consent.

**Discussion:**

The *Navigate* online decision aid has the potential to increase the choice of AS in LRPC, avoiding or delaying unnecessary radical treatments and associated side effects. In addition, *Navigate* is likely to reduce patients’ and partners’ confusion and distress in management decision-making and increase their quality of life.

**Trial registration:**

Australian and New Zealand Clinical Trial Registry ACTRN12616001665426. Registered on 2 December 2016. All items from the WHO Trial Registration Data set can be found in this manuscript.

## Background

Prostate cancer is one of the most commonly diagnosed cancers on a global scale, with incidence rates of 1.1 million during 2012 alone [1]. The vast majority of cases are detected within high-income regions such as Australia, New Zealand, Northern America and Western and Northern Europe. The high incidence rates have been attributed to the widespread use of prostate-specific antigen (PSA) testing [2]. Fortunately, the majority of prostate cancers are localised at the time of diagnosis [3]. Although early detection strategies can reduce the risk of disease progression, they can also result in overtreatment, particularly in men diagnosed with low-risk prostate cancer (LRPC).

Men diagnosed with LRPC may be offered curative treatment options, including radical prostatectomy (RP), radiotherapy (RT) and brachytherapy (BT), or may be managed with routine monitoring called active surveillance (AS). It is estimated that up to 50% of all prostate cancer cases do not require curative treatment up to 12 years post-diagnosis [4]. Indeed, evidence suggests that there is no survival benefit in providing curative treatments for LRPC at least up to 10 years after diagnosis [5, 6]. Moreover, these curative treatment options are commonly associated with both short- and long-term side effects such as erectile dysfunction, urinary and bowel incontinence and reduced quality of life [6–8]. This underscores the importance of patient involvement in decision-making regarding the commencement of curative treatment during early-stage disease [9].

AS is now recommended as a preferred management strategy for LRPC [9–13], although rates of AS uptake vary widely around the world [14]. AS is a proactive approach which aims to delay or avoid radical treatments through close monitoring of both the patient and the tumour [15]. AS is now widely accepted as an effective management strategy for LRPC, and clinicians are encouraged to offer AS to eligible patients [9, 13, 16, 17]. AS has also been shown to be the least costly option for LRPC and increased uptake of AS would lead to substantial cost-savings to the health system [18].

### Comparisons in outcomes between management options for LRPC

A recent examination of cancer mortality statistics revealed a 15-year survival rate of 95% for men with LRPC on AS [19]. In terms of age-specific outcomes for AS, 15-year cancer-specific mortality has been shown to be 5.7% for men aged 65–74 years and 10% for those over 75 [20]. Indeed, 10-year prostate cancer-specific mortality rates have been shown to be similar regardless of whether men undergo AS, RT or RP [6]. However, recent data has raised concerns about compliance with AS protocols, reporting that 75% of patients do not meet minimum requirements for PSA testing and biopsies to ensure that their cancer is adequately monitored [21].

While survival rates remain similar across management options, impacts on urinary, bowel and sexual function vary significantly. Two recent studies demonstrated significantly greater urinary incontinence and poorer sexual function in men treated with RP compared with those managed by AS [7, 22]. Importantly, men receiving AS report similar outcomes in relation to sexual, urinary and bowel function as men who are not diagnosed with LRPC [7].

### Treatment decision making in LRPC

Management decision making is difficult for men with LRPC. Decision-related distress and confusion are high [23–25], due largely to the variety of management options with no clear best choice and the fact that management decisions are highly dependent on men’s individual preferences and lifestyle [26]. For those contemplating AS, anxiety about not receiving radical (i.e. curative) treatment is common [24]. Most men do not consider all management options nor do they make decisions concordant with their personal preferences and values [27, 28]. Partners of men with LRPC also experience high decisional distress and confusion [15, 29], and some pressure their partner towards curative treatment [24, 28]. The study anticipates that both men and their partners would benefit from decision-making support, particularly regarding their understanding of the benefits and risks associated with each management option, the risk of the LRPC spreading outside the prostate and the concordance of each option with their personal values and preferences [27].

### Decision aids for LRPC

Decision aids are textual, audio, visual and web-based tools that provide evidence-based information to assist patients to consider management options within the context of their own preferences and values. Decision aids guide patients through a deliberative process of actively weighing-up the benefits/costs of available treatment options, thus enabling decision-making that is both evidence-based and considerate of patient preferences and life circumstances [30]. A 2017 Cochrane systematic review demonstrated that decision aids improve knowledge of available treatment options and outcomes, and accuracy of risk perceptions, decrease decisional conflict, increase decisional satisfaction, increase congruency between treatment choice and patient values and improve patient-clinician communication [31]. Level I evidence shows that decision aids reduce the proportion of those who choose surgery in favour of less invasive management options [13].

Several decision aids for LRPC exist, and some of these have shown promise in assisting in decision-making and reducing decisional regret [32–35]. However, two systematic reviews have concluded that none comprehensively addresses the needs of men with LRPC [26, 27]. The major deficits include incomplete or biassed content, lack of consideration of patient values, inadequate partner involvement, lack of clarification between AS and watchful waiting (WW), lack of theoretical framework, too few examples of patient experiences and inadequate systematic evaluation [26, 27]. Both reviews highlighted an urgent need for high-quality decision aids, which accurately portray AS and are developed in line with International Patient Decision Aid Standards (IPDAS) [26, 27]. Moreover, none of the currently available decision aids have been tailored to the local health care context or are easily accessible by Australian men.

### *Navigate*—an Australian online treatment decision aid for LRPC

Besides doctors, the Internet is the primary source of information for men with prostate cancer [36]. In 2017, 86% of Australian households had Internet access at home [37] while 88% of Australian adults aged 18–75 owned or had access to a smartphone [37]. Provision of an online decision aid enables access from home, promotes rapid and widespread dissemination and enables easy updating to ensure currency of content.

We have developed the *Navigate* online decision aid which presents up-to-date, unbiased information tailored to Australian men with LRPC in written, graphical and video formats. The *Navigate* decision aid was informed by a qualitative study which explored the experiences of 21 men with LRPC who had been on AS for at least 3 months, and 14 of their partners [24]. The findings confirmed that partners were highly involved in management decision-making and highlighted the need for consistent and accurate discourse surrounding LRPC diagnosis and management options. Men and their partners emphasised the need for balanced information to facilitate informed and values-based treatment decisions [24]. *Navigate* was co-designed by consumers and a multidisplinary team, is evidence-based and theoretically underpinned and complies with IPDAS criteria. Table 1 displays the key features of the website and example graphic images.

**Table 1:**
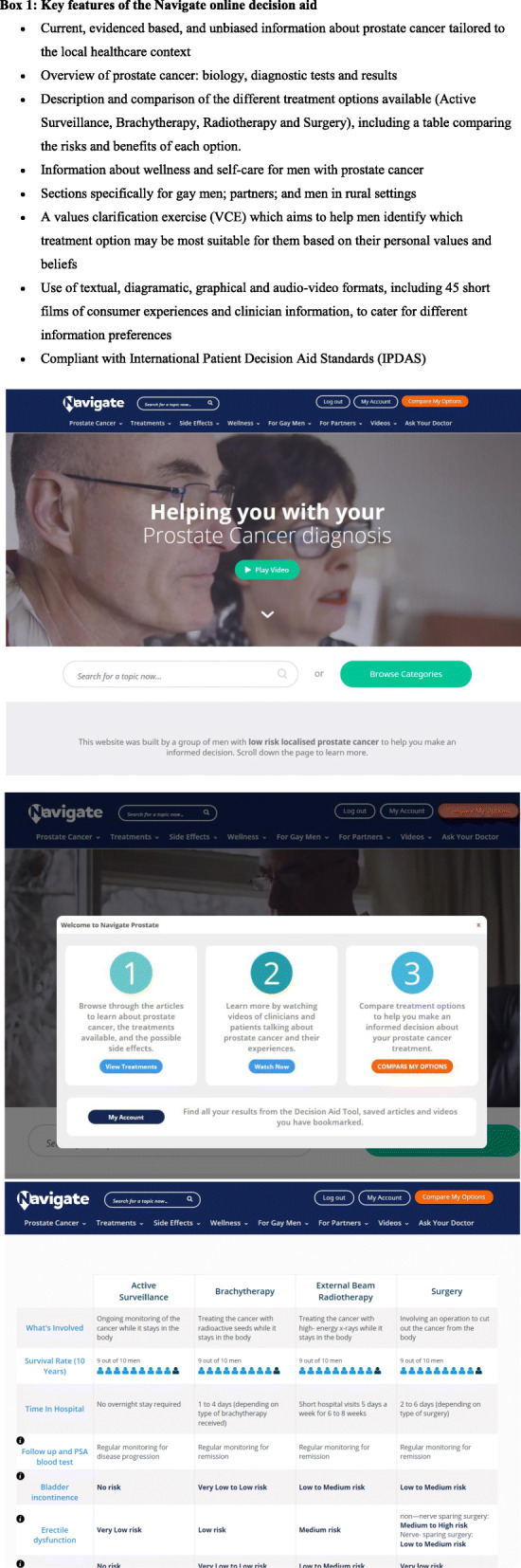
Key features of the Navigate online decision aid

## Methods

### Study aims and hypotheses

The aims of this study are to evaluate the impact of *Navigate* for men with low-risk prostate cancer and their partners on uptake of AS as a first-line management option; men’s preparedness for decision-making; men’s decisional conflict, regret and satisfaction; the quality of men’s illness communication; and men’s prostate cancer-specific quality of life. The secondary aims are to estimate study arm differences in men’s anxiety; explore the impact of *Navigate* on partner’s decisional conflict, regret and satisfaction, and quality of illness communication; assess the healthcare cost impact and cost-effectiveness of *Navigate* (economic sub-study); and determine the specific patterns of use of *Navigate* (web analytic sub-study).

Specific hypotheses are that a higher proportion of men randomised to *Navigate* will select AS as a first-line management option when compared with men randomised to the control group, and men randomised to *Navigate* will feel better prepared for decision-making, experience lower levels of decisional conflict and regret and higher levels of decisional satisfaction and report better quality of illness communication and better prostate cancer-specific quality of life when compared to men randomised to the control group.

Research questions, rather than hypotheses, were developed for secondary aims, since no specific predictions were made. Research questions relevant to the main study include:

*Do men randomised to Navigate report higher or lower levels of anxiety than men in the control group?*

*Do the partners of men randomised to Navigate experience lower levels of decisional conflict and regret, and higher levels of decisional satisfaction when compared with the partners of men randomised to the control group?*

The research question for the economic sub-study is:

*What is the cost-effectiveness of the decision-aid intervention compared with usual care for men newly diagnosed with prostate cancer?*

The research questions for the web analytic study include:

*What are the general patterns of use of the website; are there any identifiable areas for improvement from a user experience perspective?*

*Do men diagnosed with prostate cancer use the website differently compared with partners of these men?*

*What are the patterns of use across individuals relating to the values clarification exercise?*

*Do patterns of use relate to the primary and secondary outcome variables?*

### Trial design and randomisation

**Fig. 1:**
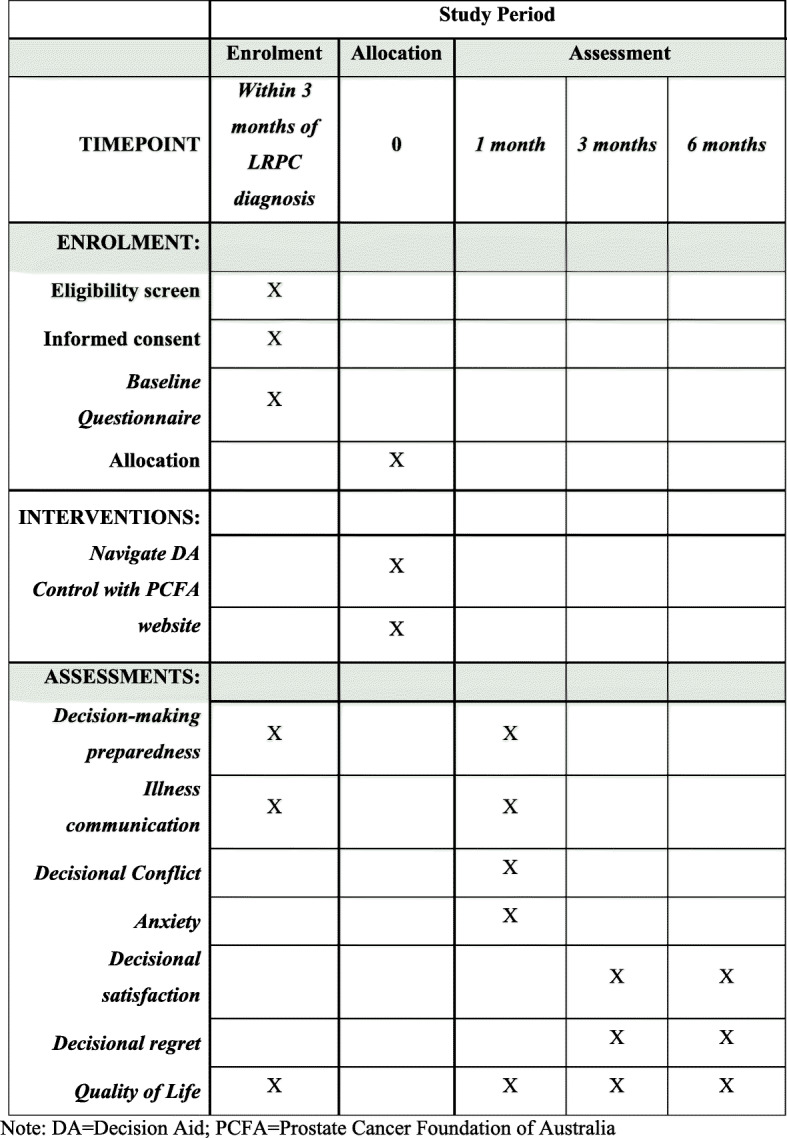
Enrolment, group allocation, intervention and assessment schedule. Note: DA, decision aid; PCFA, Prostate Cancer Foundation of Australia

This study will use a parallel group, prospective randomised controlled trial (RCT) with one baseline and three post-baseline assessments (i.e. follow-ups 1, 2 and 3), along with economic and web analytics substudies. In this study, ‘partner’ refers to the support person nominated by the patient. Both patients and their partners (if one is nominated) will be invited to participate. Following informed consent and completion of baseline study measures, participating patients and their partners will be randomly assigned to *Navigate* or the usual care arm with a one-to-one allocation, stratified by recruitment site. Randomisation will be undertaken remotely and independently by the trial coordinator using a purpose-built Microsoft Access randomisation database. After a participant (patient/partner) has been enrolled and completed baseline measures, the trial coordinator uses the randomisation database to assign the participant to the intervention or usual care arm and informs them via email of the experimental allocation. Post-baseline assessments will occur approximately 1, 3 and 6 months after consent. Participants will be informed of their allocation after completing baseline questionnaires. Researchers involved in data collection will be blinded to group allocation. Statisticians will be unblinded to allocation before preparation of the participant flow diagram and outcome analysis. Ethical approval was received from the Peter MacCallum Cancer Centre Human Research Ethics Committee (HREC16 PMCC114). Figure 1 displays the enrolment, group allocation, intervention and assessment schedule.

### Participants and study setting

Australian patients will be eligible to participate regardless of whether or not their partners participate, whereas partners are not eligible without a participating patient.

#### Eligibility criteria

Men will be eligible to participate in this trial if they are 18 years or older; have been diagnosed with LRPC in the last 3 months and have yet to make a treatment decision; have been deemed eligible for AS by their treating clinician; and have Internet access and sufficient English to complete study requirements and use the *Navigate* website. Men will be ineligible if they have a severe psychiatric or cognitive disorder or are too unwell to participate as deemed by their treating clinician, self-report, or the research team at the time of approach.

Partners are eligible to participate in this trial if they are 18 years or older, are the designated partner identified by the consenting patient and have Internet access and sufficient English to complete study requirements and use the *Navigate* website.

Participating sites include five Australian treatment centres within Victoria (Peter MacCallum Cancer Centre, Western Hospital, Cabrini Hospital, Alfred Hospital, Austin Hospital) and two in Queensland (Royal Brisbane and Women’s Hospital, Redcliffe Hospital). Men with LRPC and their partners from either public or private health care settings are also able to self-refer online to the trial.

#### Intervention

Intervention participants are emailed login details to access the *Navigate* online decision aid which is hosted on the Prostate Cancer Foundation of Australia website. Using *Navigate*, men and their partners are led through information on each treatment option for LRPC with its potential benefits and side-effects. An interactive values clarification exercise assists the men with weighing up the advantages and disadvantages of each management option to clarify what matters to them and to guide their preferences.

#### Usual care

Usual care participants are provided with minimal information to fulfil ethical obligations and emailed a link to the Prostate Cancer Foundation of Australia website page for LRPC treatments. This website provides brief information on LRPC and treatment options, but differs from the *Navigate* website in that it does not include comparisons of the pros and cons of each management option, a values clarification exercise, nor the presentation of material in graphical or multimedia formats.

### Recruitment and consent

Potentially eligible men will (i) be identified by their treating clinician/nurse or the site investigator using screening clinic lists or (ii) self-refer to the website via digital marketing strategies. The treating clinician can also refer interested patients directly to the study team. Once men and their partners provide consent and complete baseline questionnaires, they are provided with the appropriate website link as per their group allocation. Non-consenters are asked for de-identified demographic and clinical details. Attrition will be monitored and reasons for withdrawal recorded.

### Hospital recruitment and consent

Eligible men are approached after their diagnosis by research nurse/assistant, consistent with the agreement of those patients’ treating doctors. The research nurse/assistant reviews the Patient Information and Consent Form (PICF) with interested patients and patients provide online consent via the Qualtrics survey platform. Using the same consent procedure, partners are invited to participate. Consenting patients are also asked to provide written consent for the study team to obtain data from the Medicare Benefits Scheme and Pharmaceutical Benefits Scheme databases.

For patients or partners who are unsure about participating or do not have time to complete the PICF in clinic, a research team member is able to contact the patient/partner by telephone within a week to determine participation status. If the patient/partner agrees to participate, they are emailed a personalised PICF link.

### Self-referral, recruitment and consent

The study is being promoted using digital marketing and community engagement strategies (Google Ads, Facebook, social media, blogs, commentary articles). Men who self-refer to the study are asked to complete an online Expression of Interest form including the details of their treating clinician who will be contacted by the study team to confirm eligibility. If eligible, a research nurse/assistant will undertake the consent process with the patient over the telephone.

### Clinician referral, recruitment and consent

The treating clinician can also refer their patients directly the study confirming they meet the eligibility criteria and they have discussed the trial with his/her patient who is happy to be contacted. A research nurse/assistant is then available to undertake the consent process with the patient over the telephone.

### Procedure and assessments

After consent, the research team emails a link to access this questionnaire to be completed at home. *The online questionnaires were created by the research team within Qualtrics, an online program which prevents skipping questions.* A reminder telephone call is made if the study measures have not been completed within 48 h. *However, if the participant exits the form without completing the entire questionnaire, they are not followed up to obtain the remaining data.*

**Fig. 2:**
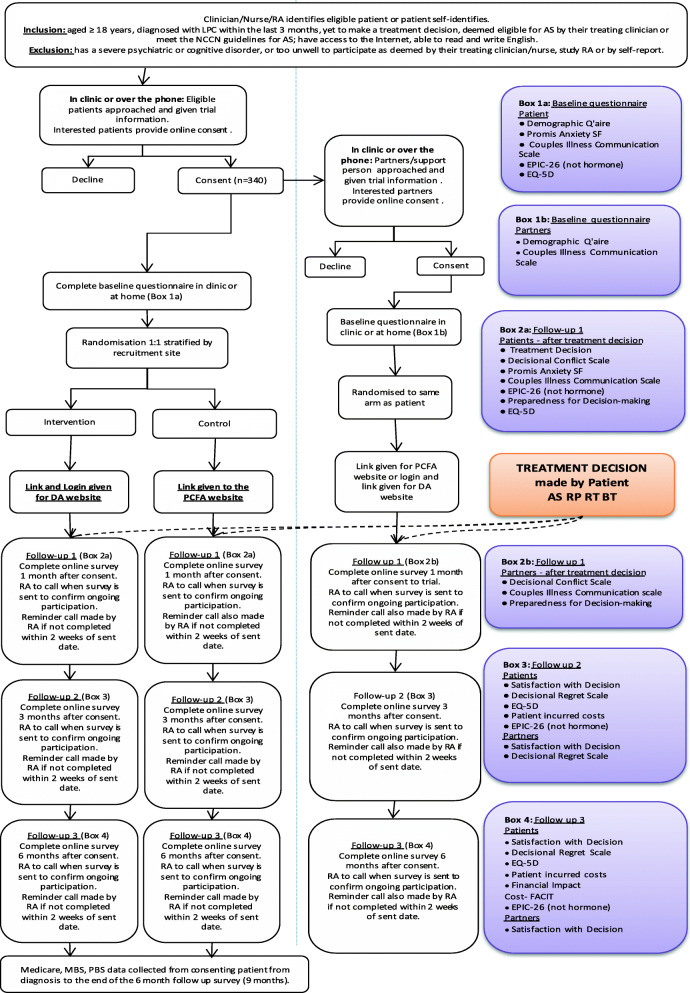
Study trial flow chart

Follow-ups 1, 2 and 3 are emailed to participants respectively at 1, 3 and 6 months post-consent. If follow-up questionnaires have not been completed within 2 weeks, a reminder telephone call is made. After completion of follow-up 3, participants are contacted via email and telephone to confirm trial completion and to thank the participant for their time. The trial flow chart is presented in Fig. 2.

### Main study measures

#### Demographic and clinical information

Demographic information collected from patients and partners includes age (in years), marital status, postcode, highest education level, occupation and ethnic origin.

Clinical information (Gleason score and PSA) is obtained from the referring site or the referring clinician or collected via a medical record audit.

### Primary outcome

The primary outcome is self-reported uptake of AS as the first-line management option for LRPC assessed as a percentage (AS or curative treatment option) at follow-up 1.

### Secondary outcomes

*Preparedness for decision-making* is assessed at follow-up 1 with the preparedness for decision-making scale (PrepDM). The PrepDM total scale measures the perceived usefulness of the decision aid in preparing the patient to communicate with his doctor(s) to make a health decision and has shown acceptable internal consistency (Cronbach’s *α* = 0.92 to 0.96), and Item Response Theory analyses demonstrated that all ten scale items function well [38].

*Decisional conflict* is assessed at follow-up 1 with the 16-item Decisional Conflict Scale (DCS). The DCS measures patients’ perceptions of uncertainty, being uninformed and unsupported and having unclear values in decision making. The DCS is suitable for use with cancer patients and has shown acceptable internal consistency (Cronbach’s *α* = 0.78–0.92), test-retest reliability (*r* = 0.81) and responsiveness (longitudinal validity) [39].

*Decisional satisfaction* is assessed at follow-up 2 and, for patients only, follow-up 3 with the 6-item Satisfaction with Decision (SWD) measure. The SWD has shown acceptable internal consistency (Cronbach’s *α* = 0.86), discriminant validity and responsiveness [31, 40].

*Decisional regret* is assessed at follow-up 2 and, for patients only, follow-up 3 with the 5-item Decisional Regret Scale (DRS). The DRS has shown acceptable internal consistency (Cronbach’s *α* = 0.81–0.92) and convergent validity with related measures (*r* = 0.31–0.60) and responsiveness [31].

The quality of men’s and partners’ *illness communication* is assessed at baseline and follow-up 1 with the 4-item Couples’ Illness Communication Scale (CICS). The CICS was adapted for use in prostate cancer populations. The original scale has shown acceptable internal consistency (Cronbach’s *α* = 0.80) and convergent validity with other measures of dyadic adjustment [41].

*Anxiety* is assessed at baseline and follow-up 1 with the 7-item PROMIS Emotional distress-Anxiety 7a short-form. The Anxiety 7a is a standardised, valid and precise measure specifically developed for use in clinical oncology research [42].

*Prostate cancer-specific quality of life* is assessed at baseline and every follow-up with the 26-item Expanded Prostate Cancer Index Composite short-form (EPIC-26). The EPIC-26 comprises four subscales: urinary, bowel, sexual and hormonal. The hormonal subscale is not relevant in this context, so will not be administered. Subscales have shown acceptable internal consistency (all Cronbach’s *α* > 0.70), test-retest reliability (all *r* > 0.69) and responsiveness when used independently [43].

### Sample size

The sample size calculation was based on 80% power, a two-sided *α* = 0.05 test, and a difference in uptake of the AS treatment option of 15%. Assuming 65% uptake in the usual care group [3] and 80% uptake in the intervention group (a conservative assumption), the required sample size is 272 patients (136 in each study arm). Our sample size calculation was performed with PASS version 16. Allowing for 10% attrition, a total sample size of 304 participants is required.

### Statistical analysis

All analyses will be performed using Stata 16. Prior to formal analysis, descriptive statistics and graphical displays will be used to identify missing and out-of-range values, assess distributional assumptions and identify outliers.

Recruitment bias will be assessed by comparing demographic and clinical characteristics of consenters and non-consenters using *t* tests (or Mann-Whitney) and chi-squared (or Fisher’s exact) tests as appropriate. Differential attrition will be assessed by comparing baseline characteristics of drop-outs and continuing participants using the same statistical tests.

### Outcome analysis

The primary analysis will follow the intention-to-treat principle and all study participants will be analysed as part of the study arm to which they were randomised in all outcome analysis. Missing data will be imputed using multiple imputation, redrawing 50 samples. Pearson’s chi squared test will be used to analyse study arm differences in the primary outcome. Linear regression will be used to analyse study arm differences for preparedness for decision-making at follow-up 1, illness communication at follow-up 1 and anxiety at follow-up. Each model will be adjusted for baseline and group assignment. Decisional conflict (follow-up 1), satisfaction (follow-ups 2 and 3) and regret (follow-ups 2 and 3) will be analysed using linear regression with group assignment as the independent covariate (equivalent to a test). The effect between groups and significance will be assessed using the beta-coefficient and *p* value corresponding to the group assignment term. Finally, prostate cancer-specific quality of life will be analysed with a random effects mixed model. The independent variables will be group assignment, time (coded 0/1) and the interaction term group assignment BY time. The effect and between groups and significance will be assessed using the beta-coefficient and *p* value corresponding to the interaction term. All results will be presented with 95% confidence intervals and a *p* value less than 0.05 (two-sided) will be deemed to be statistically significant.

### Exploratory analyses

Participants will be analysed as part of the study arm to which they were randomised in all exploratory analysis. Regression (similar to the above) will be used to analyse study arm differences in the following exploratory outcomes: men’s anxiety (at follow-up 1), partners’ illness communication with men (at follow-up 1), partners’ decisional conflict (follow-up 1), partner’s satisfaction (follow-ups 2 and 3) and partner’s regret (follow-ups 2 and 3). The effect between groups and significance will be assessed using the beta-coefficient and *p* value corresponding to the group assignment term.

### Health-economic sub-study

The aim of the economic sub-study is to assess the financial burden and cost-effectiveness of the *Navigate* online decision aid in the Australian context. This will compare the costs and patient outcomes of intervention versus usual care aligning to the goals of the planned RCT. The analysis will take a societal perspective and include Medicare data for all patient participants to assess healthcare costs to government and patient out-of-pocket expenses from participant surveys.

Health utility data will be collected at baseline and follow-ups 2 and 3. The 5-item EuroQol-5D (EQ-5D-5L) will be used, which is a standardised measure of health status developed specifically for economic evaluation and is suitable for cancer patients. It has shown acceptable internal consistency (Cronbach’s *α* = 0.71), test-retest reliability (kappa = 0.7), convergent validity (*r* > 0.49), discriminant validity and responsiveness [44, 45].

Patient health care costs will be reported by patients at follow-ups 2 and 3. These include out-of-pocket expenses for all medical services, as well as costs for travel, accommodation and income lost from interrupted employment. The Patient Costs Questionnaire (PCQ) is purpose-built for Australian men with prostate cancer [46] and includes items such as general practice and specialist visits, counselling and support services and sexual and incontinence aids. Follow-up 3 will also include the COST-FACIT questionnaire [47] capturing financial hardship and additional questions used previously by men with prostate cancer relating to early retirement and financial situation.

At the end of the data collection period, Medicare data on all services and medicines for all consenting participants will be accessed. With this data, the types and dollar amounts exchanged for health services, community services and medicines for prostate cancer and other diseases will be assessed, including medical services for privately-insured patients. The main economic outcome will be incremental cost per quality-adjusted life year for the Navigate tool versus usual care. Methods for this economic evaluation will be governed by standardised guidelines in modelling studies and economic evaluations [48, 49].

### Web analytics sub-study

Website analytics will be used to indicate preferred modes of interaction, which aspects users (participants) find most engaging and how users prefer to access information on the *Navigate* website. This provides indicative answers to specific questions, such as how comprehensively users consume the information regarding the different management options before submitting preferences on decisions. Analysis of user behaviour may lead to a better understanding of the effectiveness of the decision tool, as well as potentially identifying areas for improvement. Planned data capture includes click information, pages visited, time spent on each page, information viewed and/or downloaded and values clarification exercise responses. Since data is associated with user ID, behaviours can be linked to demographic characteristics such as age, sexual orientation, or user type (i.e. patient or partner).

Analysis will be restricted to the intervention group of the randomised study since the focus of this sub-study is the characteristics and effectiveness of the web-based decision tool. Effects to be investigated include informational value of pages (determined by number of visits and time spent), as well as complex behavioural effects such as the amount of information consumed before coming to decisions. Variance across demographic sub-groups will be measured using appropriate statistical metrics (e.g. ANOVA, chi-squared) to determine demographic-based preferences. Data mining techniques (e.g. association-rule mining) will be applied to attempt to determine other associations between behaviour characteristics (e.g. consuming certain information leading to certain decisions and/or outcomes).

### Data storage, management and future use

All data is kept in password-protected databases. Non-identifiable participant data is separated from databases linking names with participant identifiable details. Once the study is completed, this database with identifiable details will be destroyed; the remaining data will be retained indefinitely. With the exception of the Medicare data, participants who consent to this study will also be consenting to the use of their data for future unspecified research. To obtain access to the data obtained through this project, investigators will have to have their projects approved by the HREC of their host institution and by Peter Mac Cancer Centre HREC.

## Discussion

With the increase in diagnoses of LRPC [1, 50], there is an urgent need for resources to support patients and their partners in their management decision making and, specifically, to reduce the choice of potentially unnecessary radical treatments with inherent significant side effects [26, 27]. Confusion, anxiety, distress and decisional regret are common in men with LRPC [23–25] and in their partners which may be enduring [15, 24, 28, 29]. Indeed, men with LRPC and their partners express the need for unbiased information about potential benefits and risks of their management options, to help them informed decisions which are aligned their own personal preferences and values [24].

The *Navigate* online decision aid has the potential to increase the choice of AS to manage LRPC, thereby avoiding or delaying radical treatments. In addition, *Navigate* has the potential to reduce patients’ and partners’ confusion and distress in management decision making, reduce decisional regret, increase decision-making preparedness and decisional satisfaction and improve prostate cancer-specific quality of life. *Navigate* will be made available to all Australian men diagnosed with LRPC to support their management decision making.

Besides the potential ramifications on quality of life for men who receive treatment with curative intent, management of LRPC exerts a substantial financial burden to governments, hospitals and men affected by this condition which is increasing annually [18]. Approximately 25% of Australian prostate cancer patients diagnosed have LRPC [3, 11]: approximately 5500 per year. If this intervention is successful in achieving the minimal expected difference of 15%, which represents 825 men selecting AS in preference to the most common definitive treatment approach (i.e. radical prostatectomy), this could present an annual cost saving of at least AU$6.1 million to Australians (difference in average cost per patient of AU$1400 for government and AU$2300 for out of pocket) [18]. As prostate cancer diagnoses and health care costs escalate, these projected cost savings will grow. Therefore, this project has the potential to reduce both the financial costs to the government and personal costs to the large and growing population of men with LRPC.

The findings of this study will be disseminated via publications in peer-reviewed journals and by engagement with clinicians, media, government and consumers. In particular, we will promote the outcomes of this study amongst the broader medical and consumer community to improve treatment paradigms and approaches to supportive care for patients with LRPC and their partners. This will include providing the *Navigate* online decision aid as standard care for patients diagnosed with prostate cancer via the Prostate Cancer Foundation of Australia website, if ultimately proved effective.

### Trial status

The protocol version number is HREC16PMCC114_NAVIGATE_Protocol_V8_27/03/2020. Patient recruitment opened on May 2017, and it is estimated that it will cease in December 2020.

## Data Availability

The datasets generated and/or analysed during the current study are available from the corresponding author on reasonable request and in accordance with ethical restrictions imposed by the Ethics Committees that approved this study.

## Abbreviations

LRPC: Low-risk prostate cancer
AS: Active surveillance
RCT: Randomised controlled trial
RT: Radiotherapy
BT: Brachytherapy
RP: Radical prostatectomy
WW: Watchful waiting
IPDAS: International Patient Decision Aid Standards
MRC: Medical Research Council
PICF: Participant Information and Consent Form
MBS: Medicare Benefits Scheme
PBS: Pharmaceutical Benefits Scheme
DCS: Decisional Conflict Scale
SWD: Satisfaction With Decision measure
DRS: Decision Regret Scale
CICS: Couples Illness Communication Scale
EPIC-26: Expanded Prostate Cancer Index Composite short-form
EQ-5D: EuroQol-5D
