# Correction to: Navigate: a study protocol for a randomised controlled trial of an online treatment decision aid for men with low-risk prostate cancer and their partners

**DOI:** 10.1186/s13063-021-05070-6

**Published:** 2021-01-27

**Authors:** Penelope Schofield, Karla Gough, Amelia Hyatt, Alan White, Mark Frydenberg, Suzanne Chambers, Louisa G. Gordon, Robert Gardiner, Declan G. Murphy, Lawrence Cavedon, Natalie Richards, Barbara Murphy, Stephen Quinn, Ilona Juraskova

**Affiliations:** 1 Department of Psychology, Swinburne University of Technology, Melbourne, Victoria Australia; 2 Behavioural Science Unit, Peter MacCallum Cancer Centre, Melbourne, Victoria Australia; 3 Sir Peter MacCallum Department of Oncology, The University of Melbourne, Parkville, Victoria Australia; 4 Swinburne University of Technology, John Street, Hawthorn, Australia; 5 Department of Nursing, The University of Melbourne, Parkville, Victoria Australia; 6 Department of Urology, Cabrini Institute, Cabrini Health, Malvern, Australia; 7 Department of Surgery, Monash University, Melbourne, Victoria Australia; 8 Faculty of Health, University of Technology Sydney, Sydney, Australia; 9 Health and Wellness Institute, Edith Cowan University, Perth, Australia; 10 Institute for Resilient Regions, University of Southern Queensland, Springfield, Australia; 11 Menzies Health Institute Queensland, Griffith University, Southport, Australia; 12 Population Health Department, Health Economics, QIMR Berghofer Medical Research Institute, Brisbane, Queensland Australia; 13 School of Nursing, Queensland University of Technology (QUT), Brisbane, Queensland Australia; 14 School of Public Health, University of Queensland, Brisbane, Queensland Australia; 15 School of Medicine, University of Queensland, Brisbane, Queensland Australia; 16 Department of Urology, Royal Brisbane & Women’s Hospital, Herston, Queensland Australia; 17 Division of Cancer Surgery, Peter MacCallum Cancer Centre, Melbourne, Victoria Australia; 18 School of Science, RMIT University, Melbourne, Victoria Australia; 19 Department of Psychology, The University of Melbourne, Parkville, Victoria Australia; 20 Faculty of Health, Deakin University, Bundoora, Victoria Australia; 21 Department of Health Science and Biostatistics, Swinburne University of Technology, Melbourne, Victoria Australia; 22 School of Psychology, Faculty of Science, Centre for Medical Psychology and Evidence-based Decision-making (CeMPED), University of Sydney, Sydney, New South Wales Australia

**Correction to: Trials 22, 49 (2021)**

**https://doi.org/10.1186/s13063-020-04986-9**

Following publication of the original article [1], we were notified of a typo in the spelling of the 10th author name, originally spelt as “Cavdon” instead of “Cavedon” (i.e., missing “e”).

The original article has been corrected.
